# Effect of a novel sternal recumbency approach for quadratus lumborum block on isoflurane requirements in cats undergoing ovariohysterectomy

**DOI:** 10.3389/fvets.2025.1648665

**Published:** 2025-09-03

**Authors:** Fumihiko Takusagawa, Yoshimi Takusagawa, Taro Kimura

**Affiliations:** ^1^Seibozaka Animal Hospital, Tokyo, Japan; ^2^Vet Surg Tokyo, Tokyo, Japan

**Keywords:** feline, QLB, isoflurane-sparing effect, regional anesthesia, ultrasound-guided nerve block

## Abstract

**Introduction:**

This prospective, randomized clinical study with a sequential design aimed to evaluate whether a novel quadratus lumborum block (QLB) technique applied in sternal recumbency could reduce isoflurane requirements and enhance procedural safety in cats undergoing ovariohysterectomy.

**Methods:**

Thirty-five American Society of Anesthesiologists physical status (ASA-PS) I female cats, either client-owned or sheltered, undergoing ovariohysterectomy. Cats were randomly assigned to different groups to undergo either conventional QLB technique in lateral recumbency (CQLB group, *n* = 17) or novel QLB technique in sternal recumbency (NQLB group, *n* = 18). Ultrasound-guided injections were performed bilaterally, targeting the fascial plane between the quadratus lumborum and psoas minor muscles, with 0.4 mL kg^−1^ bupivacaine administered per side. Cats were premedicated with dexmedetomidine, anesthetized with propofol and isoflurane. The end-tidal isoflurane concentration (ETiso) was adjusted based on heart rate (HR), respiratory rate, and mean arterial pressure (MAP). Outcomes included total mean ETiso, phase-specific ETiso, total mean HR and MAP, rate of needle proximity to abdominal organs, rates of bradycardia, hypotension, and the need for postoperative analgesics, assessed using both the Short-form UNESP-Botucatu Multidimensional Composite Pain Scale and the Feline Grimace Scale.

**Results:**

Cats in the NQLB group demonstrated slightly lower total mean ETiso (*p* = 0.046) and significantly reduced ETiso during right ovariectomy (*p* = 0.022) and hysterectomy (*p* = 0.007) compared with cats in the CQLB group. Total mean HR and MAP did not differ between groups. Needle proximity to abdominal organs was observed in all CQLB cats but not in any NQLB cats. No bradycardia or hypotension was observed. There was no significant difference in the requirement for postoperative rescue analgesic between the groups.

**Discussion:**

The novel QLB technique demonstrated a superior isoflurane-sparing effect and safety compared with that of conventional QLB in cats. This approach may benefit cats undergoing ovariohysterectomy.

## 1 Introduction

Regional anesthesia is increasingly recognized as an important component of nociceptive management in both human and veterinary medicine, with growing evidence supporting its efficiency (1). In addition to providing analgesia, regional anesthesia is known to reduce the requirement for inhalational anesthesia in both dogs and cats (2). Given the cardiovascular and pulmonary depressive effects of isoflurane in cats (3, 4), minimizing its use can provide significant perioperative benefits. Although sevoflurane has been suggested in some studies and clinical contexts to offer cardiopulmonary advantages over isoflurane (5), isoflurane remains more widely used in veterinary anesthesia due to its lower cost and established clinical use (6).

Various regional anesthesia techniques, including epidural anesthesia (7, 8), transversus abdominis plane (TAP) block (9), and rectus sheath block (10), are commonly used for celiotomy in cats. The quadratus lumborum block (QLB), a fascial plane block involving the injection of local anesthesia around the quadratus lumborum muscle, was developed as a modification of the TAP block used in human medicine (11). Unlike TAP blocks, which primarily provide somatosensory blockade, QLB also modulates visceral nociception by spreading around visceral afferent fibers that course alongside the sympathetic trunk (12). In human medicine, QLB is widely used for abdominal procedures, including hysterectomy (13–15), cesarean section (16, 17), and nephrectomy (18). Despite its increasing use in human medicine, only a few clinical studies have investigated QLB in cats (19–21).

Existing QLB techniques used in cats, which vary according to the injection site around the quadratus lumborum muscle, are typically performed in lateral recumbency (22–24). However, this approach requires needle advancement in a ventrolateral-to-dorsomedial direction, raising concerns about the risk of injuring abdominal organs such as the kidney and spleen (25). A cadaveric study demonstrated that this technique resulted in injectate staining the retroperitoneal cavity (22). To mitigate these risks, a dorsolateral-to-ventromedial needle trajectory was proposed, but this modification exhibited poor ultrasound visibility (23). In the present study, we investigated a novel QLB approach performed in sternal recumbency. This technique employs dorsolateral-to-ventromedial needle insertion, aiming to improve ultrasound visibility while avoiding needle trajectory proximity to abdominal organs. This study aimed to evaluate the isoflurane-sparing effect, safety, and feasibility of this novel QLB technique in cats undergoing ovariohysterectomy. Preliminary clinical impressions suggested that this approach might reduce intraoperative anesthetic requirements compared to the conventional technique. Therefore, we hypothesized that the novel technique would be associated with lower end-tidal isoflurane concentrations and a reduced risk of needle proximity to abdominal organs. The primary outcome was the intraoperative end-tidal isoflurane concentration (ETiso) required to maintain anesthesia, used as a practical surrogate for anesthetic depth and clinical anesthetic requirement. Secondary outcomes included the proximity of abdominal organs to the needle trajectory, the incidence of cardiovascular complications, QLB-related complications, and postoperative pain scores.

## 2 Materials and methods

### 2.1 Animals and study design

This study followed a sequential design and was approved by the Institutional Animal Care and Use Committee of Kimura Animal Hospital (KAH2023-005). Informed consent was obtained from the owners or caregivers of all enrolled cats.

A total of 38 healthy female cats scheduled for elective ovariohysterectomy were initially enrolled. Cats were randomly assigned to either the CQLB group, in which QLB was performed via the conventional lateral recumbency approach as described by dos-Santos et al. (22), or the NQLB group, in which the novel QLB was performed in sternal recumbency. Randomization was performed by one investigator (FT) using an online list generator,^1^ which assigned numbers 1–38 to either group. Cats were then allocated to these numbers according to the order of their scheduled procedures.

Of the 38 cats enrolled, three were excluded: one due to an ASA-PS classification of II associated with respiratory disease, and two due to withdrawal of owner consent. Consequently, data from 35 cats were included in the final analysis (CQLB group: *n* = 17; NQLB group: *n* = 18).

Preanesthetic examinations, including physical examination, complete blood count, blood biochemistry, and thoracic radiography, were performed within 1 week before surgery. On the day of surgery, the cats underwent body weight measurement, Body Condition Score (BCS) assessment using a 9-point scale (26), and baseline pain assessments using the Short-form UNESP-Botucatu Multidimensional Composite Pain Scale (MCPS-SF) for Cats (27) and the Feline Grimace Scale (FGS) (28).

Cats with an American Society of Anesthesiologists physical status (ASA-PS) classification of I, scheduled for ovariohysterectomy, and either client-owned or sheltered, were enrolled in the study. Cats were excluded from the study if they met any of the following criteria: ASA-PS classification of II or greater; BCS lower than 4 or higher than 7; age younger than 4 months or older than 3 years; skin infection or any other lesion at the QLB site; pregnancy; painful conditions (MCPS-SF score ≥ 4); or prior analgesic treatment.

### 2.2 Preoperative management

Food but not water was withheld for at least 6 h before anesthesia. All cats were premedicated with dexmedetomidine (Dexdomitor 0.1 mg mL^−1^; Orion Corporation, Espoo, Finland) at 4 μg kg-^1^ intramuscularly (IM). Ten minutes later, an intravenous catheter (Surflo Flash 24-gage 3/4 inch; Terumo Corporation, Tokyo, Japan) was placed in the medial saphenous vein for the administration of anesthetic agents and electrolyte fluids. Propofol (Propoflo 28; Zoetis Inc., Parsippany, NJ, United States) was then administered intravenously for anesthetic induction. Once the swallowing reflex disappeared, 0.1 mL of 2% lidocaine (Lidocaine Hydrochloride Injection 2% “Nissin”; Nissin Pharmaceutical Co., Ltd., Yamagata, Japan) was applied around the larynx. After 30–60 s, an appropriately sized cuffed endotracheal tube (3.5–4.0 mm internal diameter, Spiral Endotracheal Tube with Stylet and Cuff; Fuji Systems Corporation, Tokyo, Japan) was placed. The propofol dose required for anesthetic induction (mg kg^−1^) was recorded. The endotracheal tube was connected to a non-rebreathing circle system (SafeSigh Non-Rebreathing System with Manometer; Vetamac, Rossville, IN, United States) integrated with an anesthetic machine (Acoma Veterinary Anesthesia Machine FO-20A; ACOMA Medical Industry Co., Ltd., Tokyo, Japan).

### 2.3 Intraoperative management

Anesthesia was initially maintained using isoflurane (Isoflurane; Viatris Healthcare G. K., Tokyo, Japan) with a vaporizer setting of 2.0, 100% oxygen, and a fresh gas flow rate of 500 mL kg^−1^ min^−1^. Spontaneous ventilation was maintained, and manual ventilation was temporarily provided if end-tidal carbon dioxide (ETCO₂) exceeded 45 mmHg, to prevent hypoventilation. Lactated Ringer’s solution (Solulact Infusion 250 mL; Terumo Corporation, Tokyo, Japan) was administered at 5 mL kg^−1^ h^−1^ throughout anesthesia. Following induction, cefazolin (25 mg kg^−1^ IV; Cefazolin Injection “Fujita”; Fujita Pharmaceutical Co., Ltd., Tokyo, Japan) and meloxicam (0.3 mg kg^−1^ subcutaneously; Inflacam® 0.5% Injection; Virbac Japan Co., Ltd., Osaka, Japan) were administered. Heart rate (HR), respiratory rate (fR), oxygen saturation of hemoglobin, ETCO₂, ETiso, and esophageal temperature were continuously measured using a multiparametric monitor (ePM12M Vet; Mindray, Shenzhen, China). ETCO₂ and ETiso were measured using a side-stream type capnometer integrated into the multiparametric monitor. The ePM12M Vet gas analyzer continuously performs automatic calibration for the measurement of the concentration of anesthetic gas, including isoflurane, as per the manufacturer’s specifications. According to the manufacturer’s recommendations, annual accuracy verification using a standard calibration gas was performed to ensure measurement reliability. Non-invasive systolic, diastolic, and mean arterial pressure (SAP, DAP, and MAP, respectively) were measured at 2.5-min intervals using an oscillometric device (PetMAP Graphic II; CardioCommand, Inc., Tampa, FL, United States) with a cuff sized to approximately 40% of the circumference of the antebrachium.

### 2.4 QLB procedures

After instrumentation, QLB was performed according to the allocated group. In both groups, 0.125% bupivacaine (Marcaine Injection 0.125%; Nissin Pharmaceutical Co., Ltd.) was administered at 1 mg kg-^1^ (total 0.8 mL kg^−1^, 0.4 mL kg^−1^ per side). All QLBs were performed by a single anesthetist (FT).

**Figure 1:**
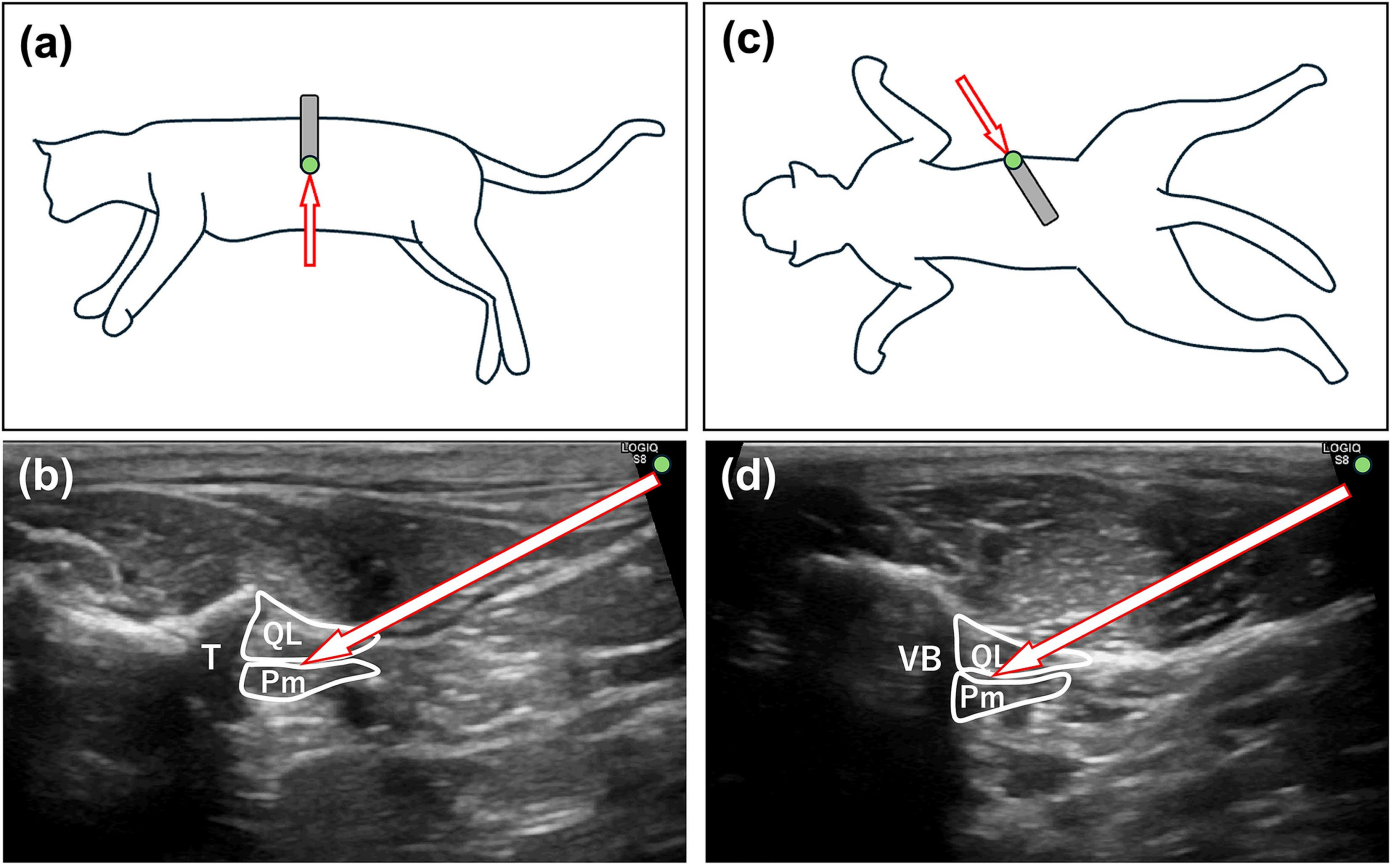
Ultrasound-guided quadratus lumborum block (QLB) in cats. **(a)** Ultrasound probe placement (gray square) and needle trajectory (arrow) in the CQLB group. A green circle indicates the position of the reference marker. **(b)** Corresponding ultrasound image in the CQLB group, with the arrow indicating the needle direction. A green circle indicates the direction of the reference marker. **(c)** Ultrasound probe placement (gray square) and needle trajectory (arrow) in the NQLB group. **A green circle indicates the position of the reference marker. **(d)** Corresponding ultrasound image in the NQLB group, with the arrow indicating the needle direction. A green circle indicates the direction of the reference marker. Pm, psoas minor muscle; QL, quadratus lumborum muscle; R, right; T, transverse process; VB, vertebral body; CQLB, conventional quadratus lumborum block; NQLB, novel quadratus lumborum block.

In the CQLB group, cats were positioned in left lateral recumbency. The hair around the second lumbar vertebra (L2) was clipped, and the skin was disinfected with 70% alcohol. A linear ultrasound (US) probe (11 L-D Linear Array Transducer; GE Healthcare, Chicago, IL, United States) connected to a US machine (LOGIQ S8; GE Healthcare) was placed perpendicular to the longitudinal axis of the cat to locate the L2 transverse process (Figure 1a), identified by counting from the last rib on the US image. The probe marker was oriented laterally (toward the right side of the cat), resulting in the lateral side appearing on the right side of the ultrasound image and the medial side on the left. The L2 transverse process and associated muscular structures were visualized in the transverse plane. Relevant anatomical structures for QLB, including the second lumbar vertebral body, transverse process, quadratus lumborum (QL) muscle, psoas minor (Pm) muscle, and the fascia between these muscles, were identified on the US image. A 22-gage, 50-mm echogenic needle (Sonolect Needle; Hakko Co., Ltd., Chikuma, Japan) was inserted at the ventral edge of the probe using the in-plane technique in a ventrolateral-to-dorsomedial direction. The needle tip was positioned within the fascia between the QL and Pm muscles (Figure 1b). Absence of blood on aspiration was confirmed, and a test injection containing 0.1–0.3 mL of injectate was performed to verify hydrodissection. After confirming hydrodissection, the remaining injectate was administered slowly. The procedure was then repeated on the contralateral side with the cat repositioned in right lateral recumbency.

Cats in the NQLB group were positioned in sternal recumbency. The hair around the L2 region was clipped bilaterally, and the skin was disinfected with 70% alcohol. The space between the transverse processes of the second and the third lumbar vertebrae was located by counting from the last rib on the US image. The probe was placed perpendicular to the longitudinal axis of the cat at the identified location and then rotated parallel to the transverse processes to identify the QL and Pm muscles (Figure 1c). The probe marker was oriented toward the right side of the cat. Accordingly, in transverse imaging, the right side of the cat (lateral) appears on the right side of the screen, and the left side (medial) on the left. A 22-gage, 50-mm echogenic needle, similar to the one used in the CQLB group, was inserted at the lateral edge of the probe using the in-plane technique in a dorsolateral-to-ventromedial direction, with the tip positioned within the fascia between the QL and Pm muscles (Figure 1d). The injection procedure was then performed as described for the CQLB group.

During needle insertion, needle visibility and proximity to abdominal organs were evaluated subjectively by the anesthetist under real-time ultrasound guidance. Needle visibility was classified as excellent when the entire needle shaft was visualized on the ultrasound image, or poor when any part of the needle was not visible. Proximity to abdominal organs was categorized as near, if any abdominal organs (e.g., kidney or spleen) were visualized along the needle trajectory, or far if no abdominal organs were observed in the vicinity of the needle path. Any complications related to the QLB technique, including unintended needle trajectories with the potential to penetrate abdominal organs, were documented.

### 2.5 Intraoperative management and ovariohysterectomy

After completion of QLB, cats were positioned in dorsal recumbency. During surgical preparation for ovariohysterectomy, the isoflurane vaporizer was initially set to achieve an ETiso of 1.0%. ETiso, HR, fR, and MAP were recorded 10 min after completion of QLB (T0) and subsequently every 2.5 min throughout anesthesia. Throughout anesthesia, ETiso was adjusted as follows: increased by 0.2% if HR, fR, or MAP exceeded 20% of T0; increased by 0.5–1.0% if these parameters exceeded 30% of T0 or if the palpebral reflex, increased jaw tone, or purposeful movement were observed; decreased by 0.2% if MAP dropped below 70 mmHg; or the vaporizer setting was decreased by 0.5–1.0% if MAP dropped below 60 mmHg. If ETiso exceeded 2.8%, which was predetermined as approximately 1.5 times the minimum alveolar concentration (MAC) of isoflurane in cats (1.87%) based on previous studies (29), remifentanil (0.01 mg kg^−1^ h^−1^ IV; Ultiva Injection 2 mg; Janssen Pharmaceutical K. K., Tokyo, Japan) was administered as rescue analgesia, In all such cases, buprenorphine was also administered postoperatively for ongoing analgesic support. The occurrence of intraoperative rescue remifentanil administration was recorded, and data collected after its administration were excluded from further analysis. If MAP remained below 70 mmHg despite reducing ETiso to below 0.6% or until a light plane of anesthesia was observed, indicated by explicit palpebral reflex, increased jaw tone, or purposeful movement, appropriate interventions were initiated. These included a bolus administration of lactated Ringer’s solution (3 mL kg^−1^ over 5 min), atropine (0.04 mg kg^−1^ IV; Atropine Sulfate Injection 0.5 mg “Nipro”; Nipro ES Pharma Co., Ltd., Osaka, Japan), dopamine (0.005 mg kg^−1^ min^−1^ IV; Dopamine Hydrochloride Intravenous Infusion 100 mg “NP”; Nipro Corporation, Osaka, Japan), or ephedrine (0.1 mg kg^−1^ IV; Ephedrine “Nagai” Injection 40 mg; Nichi-Iko Pharmaceutical Co., Ltd., Toyama, Japan). Cases requiring pharmacological intervention for hypotension were excluded from data analysis. Intraoperative cardiovascular depression, defined as bradycardia (HR < 100 bpm) or hypotension (MAP < 70 mmHg requiring drug intervention), was documented.

**Figure 2:**
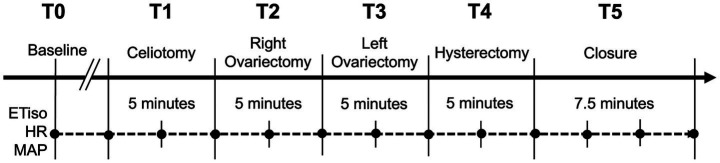
Timeline of surgical periods and corresponding elapsed times for ovariohysterectomy performed in this study. Solid black circles indicate the time points at which ETiso (end-tidal isoflurane concentration), HR (heart rate), and MAP (mean arterial pressure) are recorded.

Ovariohysterectomy was initiated 2.5–5.0 min after recording of T0 and performed by a single surgeon (YT) who was blinded to group allocation using a standardized technique (30). The surgery was divided into five periods: T1 (celiotomy), T2 (left ovariectomy), T3 (right ovariectomy), T4 (hysterectomy), and T5 (closure), with corresponding durations of 5, 5, 5, 5, and 7.5 min, respectively, as shown in Figure 2. During surgery, a forced-air warming blanket (Bair Hugger; 3 M Company, St. Paul, MN, United States) was used to maintain esophageal temperature above 36.5 °C. After surgery, isoflurane was discontinued, and the cats were extubated once the swallowing reflex was restored.

### 2.6 Postoperative pain and QLB-related complications

Postoperative pain was assessed by the anesthetist (FT) using MCPS-SF and/or FGS at 1, 2, 4, and 8 h after extubation. If either or both pain scores reached 4 or higher, intramuscular buprenorphine (0.02 mg kg^−1^; Lepetan Injection 0.2 mg; Otsuka Pharmaceutical Co., Ltd., Tokyo, Japan) was administered as rescue analgesia, and subsequent data were excluded from analysis. Pain scores were reassessed 30 min after the administration of the rescue analgesic, and additional doses were administered as needed. The number of cats in each group requiring postoperative rescue analgesia, along with the time to its first administration when applicable, was recorded.

Postoperative complications related to QLB, such as postoperative ataxia, needle-site bleeding, or signs of local anesthetic systemic toxicity, were monitored and documented if observed.

### 2.7 Statistical analysis

The sample size was calculated during the planning phase using G*Power version 3.1.9.7 (31), based on an expected 20% difference in mean end-tidal isoflurane concentration (ETiso), with a standard deviation (SD) of 0.34 derived from our internal pilot data. Assuming a two-sided test with 80% power and a 5% significance level, a minimum of 17 cats per group was required. To account for a potential dropout rate of approximately 10%, 19 cats were included in each group.

The total mean ETiso, HR, and MAP were calculated as the area under the curve (AUC), determined using the trapezoidal rule, and divided by the total surgery time. Similarly, the mean ETiso for each surgical period (T1–T5) was calculated as the AUC, determined using the trapezoidal rule, and divided by the respective procedure time. The Shapiro–Wilk test was used to examine the normality of data distribution. Continuous variables are expressed as mean ± SD if normally distributed and as median (range) if non-normally distributed. Rates of postoperative rescue analgesia requirement were expressed as proportions.

The total mean ETiso, HR, and MAP were compared between groups using a *t*-test. The mean ETiso during each procedure was compared using repeated measures analysis of variance, followed by Tukey’s *post hoc* test for pairwise comparisons at each time point. Postoperative incidence of rescue analgesia was compared using Kaplan–Meier survival analysis and the log-rank test. A *p*-value of <0.05 was considered statistically significant. All statistical analyses were performed using EZR version 1.68 (Saitama Medical Center, Jichi Medical University, Saitama, Japan).

## 3 Results

Demographic data, including age, body weight, BCS, breed distribution, and propofol dose for anesthetic induction, are summarized in Table 1. The groups were comparable in terms of all demographic variables. Total mean HR was 137 ± 12.3 in the CQLB group and 138 ± 7.22 in the NQLB group, with no statistically significant difference (*p* = 0.823; 95% CI: −7.65 to 6.13). Total mean MAP was 83 ± 6.91 mmHg in the CQLB group and 87.1 ± 9.12 mmHg in the NQLB group, also showing no significant difference (*p* = 0.141; 95% CI: −9.73 to 1.45).

**Table 1:** Demographic data obtained from 35 cats (CQLB group: *n* = 17; NQLB group: *n* = 18).

Group	*n*	Age (months)	Body weight (kg)	BCS	Breed	Propofol dose (mg kg^−1^)
CQLB	17	5 (4–12)	2.00 (1.65–3.30)	4 (4–6)	Mixed Breed (14) Munchkin (1) Scottish Fold (1) Siberian (1)	6.52 ± 0.92
NQLB	18	5 (4–12)	2.22 (1.65–4.12)	4 (4–6)	Mixed Breed (13) Siberian (2) Abyssinian (1) Kinkalow (1) Norwegian Forest Cat (1)	6.09 ± 1.31

CQLB: group that undergoes conventional quadratus lumborum block (QLB) in lateral recumbency; NQLB: the group that undergoes novel QLB in sternal recumbency; BCS: body condition score.

Data are presented as median (range) or mean ± SD, as appropriate. Breed data are presented with the number of cats (n) in parentheses.

**Figure 3:**
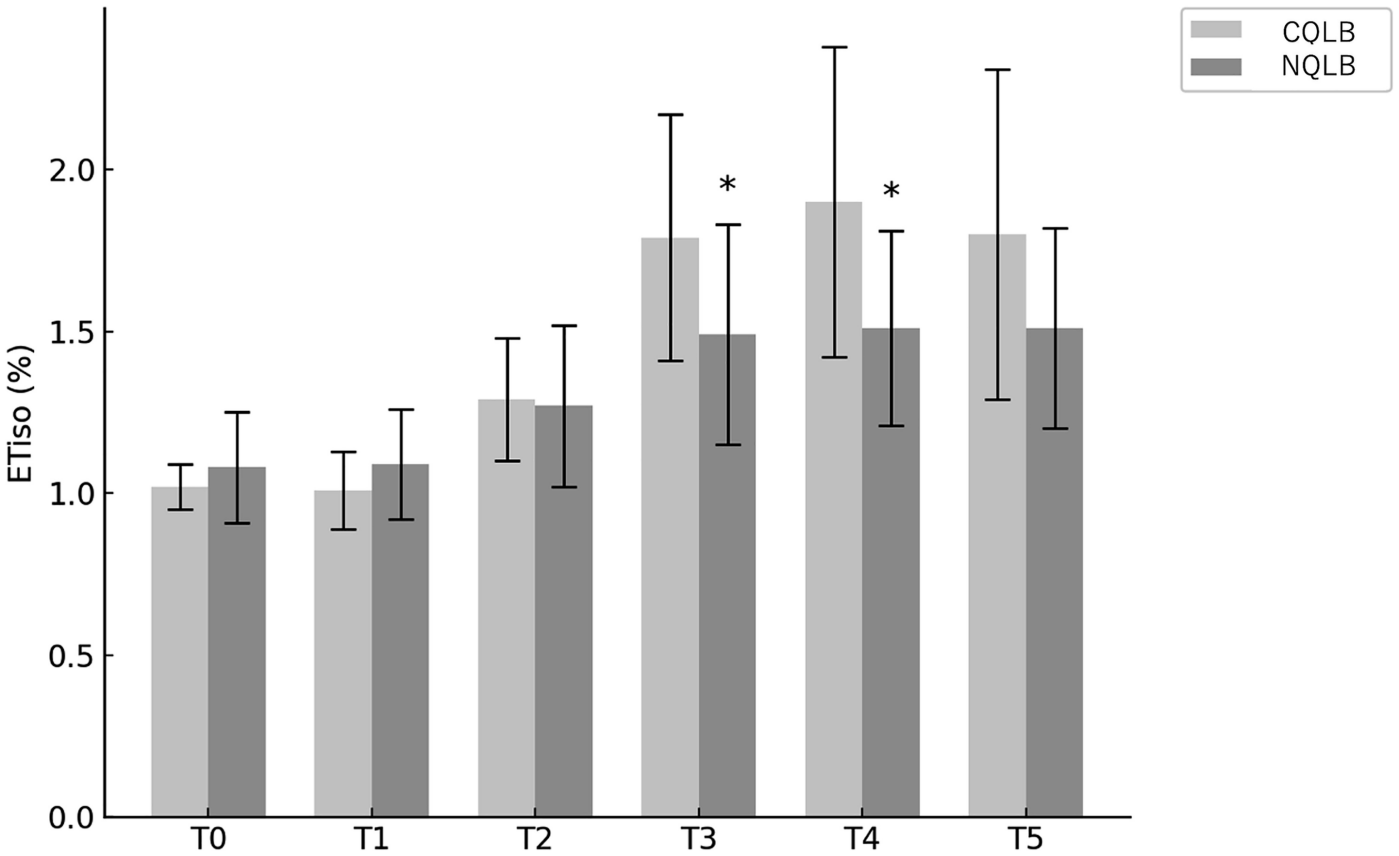
Mean end-tidal isoflurane concentration (ETiso) was recorded in 35 cats (CQLB group: *n* = 17; NQLB group: *n* = 18) enrolled in the study. Bars represent the mean ETiso, and whiskers indicate the standard deviation. The symbol (*) denotes statistically significant differences between groups. CQLB: refers to the conventional quadratus lumborum block (QLB) performed in lateral recumbency, and NQLB refers to the novel QLB performed in sternal recumbency. T0 represents the baseline, while T1 through T5 correspond to specific surgical stages: T1, celiotomy; T2, left ovariectomy; T3, right ovariectomy; T4, hysterectomy; and T5, closure.

The total ETiso was 1.39% ± 0.22 and 1.58% ± 0.31% in the NQLB and CQLB groups, respectively, with a significantly lower value observed in the NQLB group (*p* = 0.046; 95% CI: 0.00–0.37). The mean ETiso at each surgical period is shown in Figure 3. The mean ETiso in the NQLB group was significantly lower than that in the CQLB group at T3 (1.49% ± 0.34% vs. 1.79% ± 0.38%; *p* = 0.022; 95% CI: 0.04–0.54) and T4 (1.51% ± 0.30% vs. 1.90% ± 0.48%; *p* = 0.007; 95% CI: 0.11–0.66), whereas no significant differences were observed at other surgical periods (T0: *p* = 0.228; 95% CI: −0.14–0.04, T1: *p* = 0.109; 95% CI: −0.18–0.02, T2: *p* = 0.819; 95% CI: −0.14–0.17, T5: *p* = 0.052; 95% CI: −0.00–0.58).

In both groups, the visibility of the needle tract was rated as excellent in all cats, as the entire shaft was visualized on ultrasound images. In the CQLB group, at least one abdominal organ—such as the right kidney, spleen, or aorta—was visualized adjacent to the needle trajectory in all cats and thus categorized as “near.” In contrast, no abdominal organs were detected along the needle trajectory in the NQLB group, and all cases were classified as “far.” No intraoperative bradycardia or hypotension requiring drug treatment was observed in either group. Additionally, no complications related to the QLB technique were observed during anesthesia.

**Figure 4:**
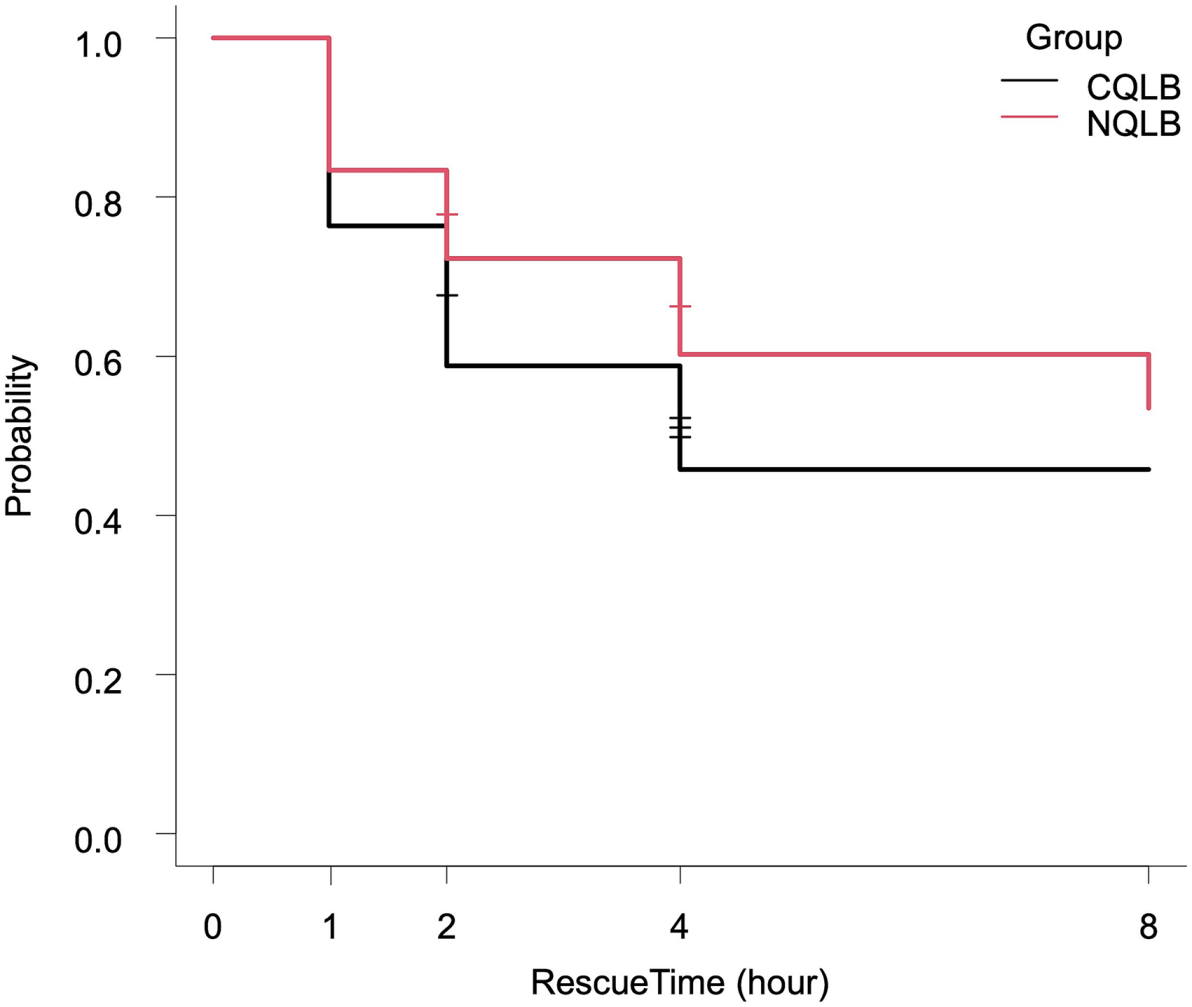
Kaplan–Meier curve depicting the rate and timing of postoperative rescue analgesic requirement in 35 cats (CQLB group: *n* = 17; NQLB group: *n* = 18). The x-axis (RescueTime) indicates the timing of postoperative assessments, while the y-axis represents the probability of not requiring postoperative rescue analgesia. Horizontal short lines on each curve represent censoring, with the number of lines indicating the number of censored cats. CQLB refers to the conventional quadratus lumborum block (QLB) performed in lateral recumbency, and NQLB refers to the novel QLB performed in sternal recumbency.

The proportion of cats requiring postoperative rescue analgesia was 9/17 (52.9%) in the CQLB group and 8/18 (44.4%) in the NQLB group. Figure 4 presents the Kaplan–Meier curve depicting the probability of not requiring rescue analgesia over time in both groups. No significant difference in the requirement for postoperative rescue analgesia was observed between the CQLB and NQLB groups (*p* = 0.505). Among the cats requiring rescue analgesia, administration was triggered by the FGS alone in 5/9 cats in the CQLB group and 7/8 cats in the NQLB group, and by both the FGS and the MCPS-SF in 4/9 cats in the CQLB group and 1/8 cats in the NQLB group. All cats that received rescue analgesia required only a single dose. Postoperative pain assessment was censored for 4/17 (23.5%) cats in the CQLB group and 2/18 (11.1%) cats in the NQLB group due to aggressive behavior. These censored cats were administered buprenorphine (0.02 mg kg^−1^ IM) regardless of their final pain scores. No clinically recognized postoperative complications related to the QLB technique were observed.

## 4 Discussion

The results of this study suggest that the novel QLB technique performed in sternal recumbency was associated with a lower isoflurane requirement compared to the conventional technique in lateral recumbency. The absence of complications, such as cardiovascular depression or abdominal organ penetration, further supports the safety of this technique.

In the present study, ovariohysterectomy was selected as a representative mid-to-lower abdominal surgery due to its involvement of a wider area of visceral innervation compared to ovariectomy, allowing for a more comprehensive evaluation of the novel QLB’s antinociceptive effects.

The isoflurane-sparing effect, observed particularly during right ovariectomy and hysterectomy performed using the novel technique, may have occurred primarily as a result of antinociceptive efficacy. Reducing the requirement for isoflurane is beneficial for cats, because this agent has been reported to induce dose-dependent cardiopulmonary depression (3). This reduction may contribute to improved hemodynamic stability during anesthesia, supporting the perioperative advantages of this novel technique.

The differences in isoflurane requirements between the groups during certain phases of the procedure in the present study may be explained by variations in local anesthetic distribution. The novel dorsolateral-to-ventromedial needle trajectory may improve local anesthetic spread to the sympathetic trunk and visceral afferents compared to the conventional approach. However, a previous cadaveric study (23) reported no significant difference in injectate spread in dorsolateral-to-ventromedial and ventrolateral-to-dorsomedial lateral recumbency. This difference in findings may be attributed to the distinct nature of cadaveric and live animal studies, as factors such as tissue compliance and perfusion can influence injectate distribution *in vivo* (32, 33). Additionally, the needle bevel faced the ventrolateral direction in the dorsolateral-to-ventromedial approach in the previous cadaveric study (23), while it faced medially in the present study. This difference in bevel direction may partly explain the discrepancy in results between the two studies, as seen in other fascial-plane blocks in humans (33, 34). Future studies focusing on needle direction, bevel orientation, and the spread of the injectate in QLB are warranted.

Interestingly, we failed to detect a superior isoflurane-sparing effect in the novel QLB during left ovariectomy. No previous studies have reported differences in injectate spread between the left and right sides for QLB in any species. However, local anesthetic distribution may differ between sides *in vivo*, as anatomical variations in the QLB target have been observed between the right and left sides in human studies (35). Additionally, the fixed 5-min duration for ovariectomy in the present study may have been too short to capture detectable changes in ETiso during left ovariectomy.

A progressive increase in ETiso was observed from T3 onward in both groups, which may reflect the waning effect of the α2-agonist used for premedication, as its peak plasma concentration (Tmax) is reached at approximately 26 min and declines thereafter (36). Although it is difficult to determine whether the changes in ETiso during each surgical phase were due to the block or other factors (e.g., nociceptive stimuli or anesthetic effects), both groups followed the same anesthetic protocol. Therefore, such systemic influences were equally present and are unlikely to explain the between-group differences observed during these phases.

A feline cadaveric study on QLB using the ventrolateral-to-dorsomedial needle insertion technique in lateral recumbency reported that the colorant stained the retroperitoneal cavity in all cadavers (22). The same authors later investigated the dorsolateral-to-ventromedial technique in lateral recumbency to reduce the risk of penetrating abdominal organs; however, poor needle visibility was reported, likely due to interference from surrounding anatomical structures (23). In contrast, the novel QLB technique used in the present study offered excellent needle visibility and a trajectory distant from abdominal organs, minimizing the perceived risk of organ penetration. These features likely contributed to the operator’s impression of enhanced procedural safety. In this study, the proximity between the needle and abdominal organs was assessed subjectively, as simple absolute distance does not necessarily reflect the true safety profile. Future investigations are warranted to objectively evaluate the safety of this novel technique using more sophisticated and standardized methods.

A feline cadaveric study showed that both QLB with dorsolateral-to-ventromedial and ventrolateral-to-dorsomedial needle insertion in lateral recumbency showed similar injectate spread patterns to the lumbar nerves innervating the abdominal wall (23). Consistent with these findings, similar isoflurane requirements during celiotomy and closure were observed for both QLB techniques in the present live animal study. Given the absence of significant differences in isoflurane requirements between groups during these phases, the total difference in isoflurane requirements appears relatively small.

In previous studies using QLB in cats, the incidence of hypotension was very low (20, 21). Consistent with these findings, both QLB techniques in the present study provided hemodynamic stability. A previous clinical study comparing the effects of QLB and sacrococcygeal epidural anesthesia (SCE) in cats undergoing ovariectomy reported a significantly lower proportion of intraoperative hypotension in the QLB group compared with that in the SCE group (19). These positive hemodynamic profiles support the selection of QLB as a beneficial option.

Postoperative pain was assessed using both the MCPS-SF and FGS to minimize the risk of incomplete assessments, given that no exclusion criteria regarding behavior were applied. However, in the study, some cats could not be assessed with both scales due to their aggressive behavior. Hence, it was necessary to censor the postoperative pain assessments.

Postoperative rescue analgesic requirements in this study were higher than those reported in previous studies (19, 20), which observed considerably lower rates of postoperative analgesic use (5.6–20.0%). This discrepancy may be attributed to differences in the concentration of local anesthetic used for QLB. In this study, 0.125% bupivacaine was used. The efficacy of this low concentration for interfascial plane blocks has not been specifically studied in veterinary medicine. However, the dose was chosen based on recommendations for cats (37, 38). Additionally, the selected dose considered the relatively high injection volume (0.4 mL kg^−1^ per side) required for adequate fascial plane distribution. The use of lower concentrations is to minimize the risk of systemic toxicity while still achieving effective analgesia, though it may have contributed to the higher rate of postoperative rescue analgesic use observed in this study. Additionally, previous studies (19–21) administered both methadone and dexmedetomidine as premedication, whereas only dexmedetomidine was used in the present study. Methadone provides effective postoperative analgesia in cats undergoing ovariohysterectomy (39), and this may have contributed to differences in postoperative lock analgesic requirements between studies. Furthermore, as observed in previous studies (40), the aggressive behavior of some cats in this study may have compromised the reliability of pain assessments. Further research is needed to evaluate the postoperative analgesic efficacy of novel QLB techniques, particularly in a cat population with moderate behavior, to minimize behavioral bias in pain assessment, and to explore broader clinical benefits such as postoperative well-being and recovery.

The present study had some limitations. First, the anesthetist, who also performed postoperative pain assessments, was not blinded to group allocation, potentially introducing bias. However, intraoperative ETiso adjustments were based on objective cardiopulmonary parameters, which likely minimized bias during data collection. The subjective nature of the postoperative pain scale may have affected reliability, and future studies should include blinded assessors to further reduce bias. Second, the novel QLB method used in this study has not been evaluated in cadaveric studies, leaving its sonoanatomy unverified against gross anatomy and its injectate spread unassessed using colorants or contrast agents. Similarly, while the injection site and volume of the local anesthetic were determined based on previous studies (19, 21, 22), the optimal parameters for QLB in cats remain unidentified. Further research is needed to investigate the sonoanatomy, injectate distribution, and optimal injection site and volume for this technique. Third, this study lacked a control group (no QLB), limiting the evaluation to a comparison of the efficacy of the two techniques. A negative control group was excluded due to ethical concerns, as the pilot study showed that cats without QLB required an ETiso exceeding 2.8%. While prior studies demonstrated the efficacy of QLB in lateral recumbency (19–21), the novel technique used in this study achieved an even greater reduction in isoflurane requirements, suggesting enhanced efficacy despite the absence of a negative control group. Fourth, intraoperative changes in cardiovascular parameters induced by noxious stimuli were managed by adjusting isoflurane concentration. Although, currently recommended practices suggest addressing such changes with antinociceptive interventions, such as opioid administration, the authors opted to titrate isoflurane in response to nociceptive signs due to concerns about adverse effects reported in cats receiving potent opioids—particularly fentanyl and remifentanil—including dysphoria, increased locomotor activity, tachycardia, and acid–base disturbances (41, 42). Furthermore, the 2.8% ETiso threshold used to trigger intraoperative rescue analgesia in the present study was based on a MAC of 1.86% in cats reported by Belli et al. (27). As reported, MAC values vary substantially among studies, establishing a definitive cut-off for adequate surgical anesthetic depth remains challenging. Future studies are warranted to compare opioid-based protocols with the novel QLB technique and to evaluate the potential opioid-sparing effect of this approach under protocols allowing timely systemic analgesic rescue, including investigation of its ability to reduce opioid-related adverse effects. Finally, the sample size estimation in this study was based on the total ETiso requirement as the primary outcome. Therefore, the interpretation of other analyses, including mean ETiso at each surgical period, mean total HR and MAP, and postoperative rescue analgesic requirements, is limited. Additionally, the relatively small sample size (n = 35) may limit the generalizability of these findings to broader clinical settings. Any significant differences observed in these secondary outcomes may not accurately reflect the true effect size owing to the possibility of an overestimated effect. Conversely, non-significant differences may have resulted from insufficient statistical power (type II error), potentially obscuring clinically relevant findings. Future studies with larger sample sizes specifically designed to evaluate these secondary outcomes and enhance the generalizability of the findings are warranted.

## 5 Conclusion

This study demonstrated that the novel quadratus lumborum block (QLB) technique, performed in sternal recumbency with a dorsolateral-to-ventromedial needle trajectory, was associated with a reduced isoflurane requirement and appeared to enhance needle placement safety when compared to the conventional lateral recumbency approach in cats undergoing ovariohysterectomy. However, given the limitations of the present study—including lack of blinding, small sample size, and the sole focus on intraoperative isoflurane concentrations—these results should be interpreted with caution.

Future investigations are warranted to evaluate the postoperative analgesic efficacy of this technique, optimize the injection site and volume, and assess the spread of local anesthetic agents in both cadaveric and live feline models.

Overall, these findings may contribute to the refinement of regional anesthetic protocols in feline surgery.

## Data Availability

The raw data supporting the conclusions of this article will be made available by the authors, without undue reservation.

## Footnotes

^1^ https://www.random.org/lists/
